# Epigenetic modification regulates the ligamentum flavum hypertrophy through miR-335-3p/SERPINE2/β-catenin signaling pathway

**DOI:** 10.1186/s11658-024-00660-z

**Published:** 2025-01-03

**Authors:** Yongzhao Zhao, Shuai Jiang, Longting Chen, Qian Xiang, Jialiang Lin, Weishi Li

**Affiliations:** 1 Department of Orthopaedics, Peking University Third Hospital, Peking University, No.49 NorthGarden Road, Haidian District, Beijing, 100191 Beijing China; 2 Beijing Key Laboratory of Spinal Disease Research, Beijing, China; 3 Engineering Research Center of Bone and Joint Precision Medicine, Ministry of Education, Beijing, China

**Keywords:** Lumbar spinal stenosis, Ligamentum flavum hypertrophy, Fibrosis, Epigenetic modification

## Abstract

**Background:**

Epigenetic modifications have been proved to play important roles in the spinal degenerative diseases. As a type of noncoding RNA, the microRNA (miRNA) is a vital class of regulatory factor in the epigenetic modifications, while the role of miRNAs in the regulation of epigenetic modifications in ligamentum flavum hypertrophy (LFH) has not been fully investigated.

**Methods:**

The miRNA sequencing analysis was used to explore the change of miRNA expression during the fibrosis of ligamentum flavum (LF) cells caused by the TGF-β1 (10 ng/ml). The downregulated miRNA miR-335-3p was selected to investigate its effects on the fibrosis of LF cells and explored the accurate relevant mechanisms.

**Results:**

A total of 21 miRNAs were differently expressed during the fibrosis of LF cells. The downregulated miR-335-3p was selected for further investigation. MiR-335-3p was distinctly downregulated in the LFH tissues compared to non-LFH tissues. Overexpression of miR-335-3p could inhibit the fibrosis of LF cells. Further research showed miR-335-3p prevented the fibrosis of LF cells via binding to the 3′-UTR of SERPINE2 to reduce the expression of SERPINE2. The increased SERPINE2 expression might promote the fibrosis of LF cells via the activation of β-catenin signaling pathway to promote the transcription of fibrosis-related genes (ACTA2 and COL3A1).

**Conclusions:**

Our results revealed that miR-335-3p prevented the fibrosis of LF cells via the epigenetic regulation of SERPINE2/β-catenin signaling pathway. The epigenetic regulator miR-335-3p might be a promising potential target for the treatment of LFH.

**Graphical Abstract:**

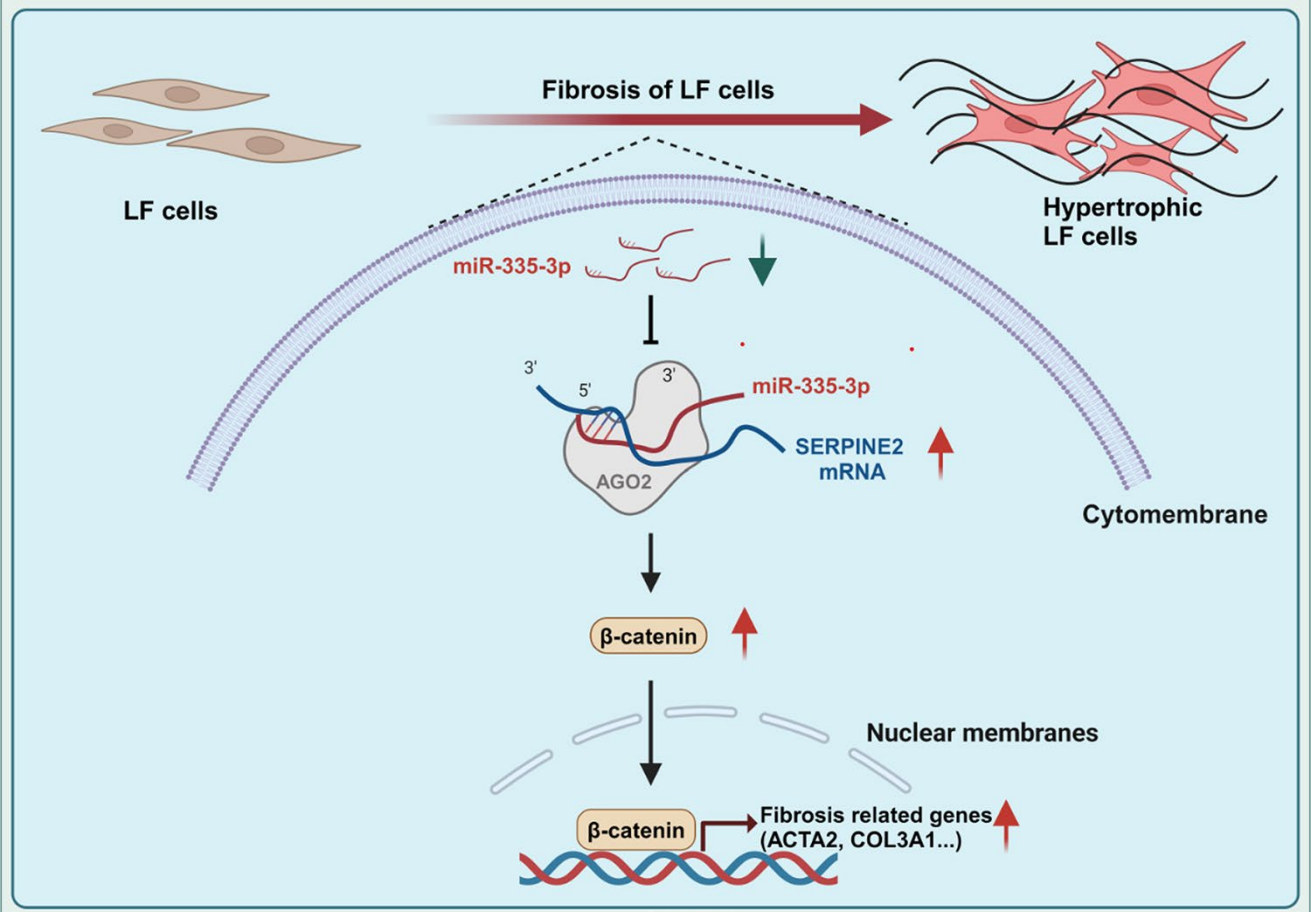

**Supplementary Information:**

The online version contains supplementary material available at 10.1186/s11658-024-00660-z.

## Background

Lumbar spinal stenosis (LSS) is the most common cause of low back pain and gait abnormality among the elderly, which seriously affects the quality of life and brings a heavy economic burden to the individuals [1]. Ligamentum flavum hypertrophy (LFH) is one of the most common reasons for LSS. During the process of LFH, the degenerative ligamentum flavum gradually becomes thicker, and finally reached the diagnostic criteria with the thickness greater than 4 mm. The hypertrophic LF will compress the spinal cord and nerve roots, leading to the LSS. LFH can result in the neurological dysfunctions, such as low back pain, intermittent claudication, and the decreased muscle strength of lower limbs [1, 2].

Ligamentum flavum (LF) is a very important spinal ligament, which participates in the formation of posterior spinal column. The main functions of LF include maintaining the stability of the spine and protect the spinal cord. The LF mainly consists of 80% elastic fibers and 20% collagenous fibers under normal physiological conditions [3]. However, in the pathological state, the LF cells, a type of fibroblasts, will transform into the activated type, named as myofibroblasts. As a fibrotic phenotype, the myofibroblasts secrete lots of collagens, which results in the degradation of elastic fibers and accumulation of collagen fibers [2]. Many cytokines have been demonstrated to contribute to the fibrotic process in fibrotic diseases, and TGF-β1 is the most import one for the fibrosis, and has been widely used to construct the cell fibrosis model, including LF cells [4, 5].

Epigenetic modification refers to the reversible and heritable changes in gene function without any alteration of DNA sequences, including regulation of noncoding RNA, DNA methylation, histone modification, and RNA modification [6–11]. MicroRNAs (miRNAs) refer to a family of noncoding RNA with about 20 nucleotides length, which regulate the gene expression at the post-transcriptional level by interacting with the 3′-untranslated region (UTR) of targeted mRNA [12, 13]. MiR-335-3p, as a star miRNAs, has been proved to be involved in several diseases, such as pulmonary arterial hypertension [14] and osteoarthritis [15]. However, the epigenetic modification mediated by the miR-335-3p in the LFH has not been investigated to date. Serpin family E member 2 (SERPINE2), also known as protease nexin 1, is the member 2 of the serpin peptidase inhibitor clade E and serves as a protease inhibitor [16, 17]. Previous studies reported that SERPINE2 might play an important role in fibrotic diseases, such as myocardial fibrosis [17, 18] and pulmonary fibrosis [19]. But the accurate role of SERPINE2 in the fibrosis of LF cells has not been explored.

In this study, we conducted the microRNA sequencing analysis of LF cells treated with TGF-β1 and found that miR-335-3p was significantly downregulated during the fibrosis of LF cells. Then, we demonstrated that miR-335-3p regulated the fibrosis of LF cells though SERPINE2/β-catenin signaling pathway. Our results showed miR-335-3p played an important role in the LFH in the manner of epigenetic regulation.

## Materials and methods

### Human LF samples

This study has been proved by the Institutional Research Ethics Committee of the Peking University Third Hospital. Informed consent was obtained from all patients who participated in the study. A total of LF sample tissues collected from 24 patients with LFH or lumbar disc herniation at L4/5 level were used in this study, and the details of included patients were listed in Supplementary Table 1. The diagnosis of LFH caused by LF hypertrophy was conducted on the basis of the combination of imaging examination results and clinical symptoms. For the imaging examinations, the magnetic resonance imaging (MRI) was generally used, and the thickness of LF > 4 mm was usually regarded as the diagnostic criteria [1, 20]. For the measurement of LF width on MRI, the width of LF were measured three times by two senior surgeons blind to this research, and the average thickness was used for analysis [20].

### Cell cultures

LF samples were acquired from patients who received the surgery for lumbar disc herniation. The LF samples were carefully minced, and then digested using 0.2% type I collagenase (#17100017, Gibco, USA) at 37 °C for 1.5 h. After the digestion, the LF tissues were washed with PBS (#10010023, Gibco, USA) and transferred to cell culture bottle (#707001, NEST, China) containing DMEM with 10% fetal bovine serum) (#A5256701, Gibco, USA), 100 mg/ml streptomycin, and 100 U/ml penicillin (#15140148, Gibco, USA). The samples were then incubated at 37 °C in a 5% CO_2_ cell incubator. Cells at three to five passages were obtained for subsequent cell experiments. To induce the fibrosis of LF cells, the recombinant protein TGF-β1 (#HY-P70543, MedChemExpress, USA) (10 ng/ml) was used to treat the LF cells. To inhibit the β-catenin signaling pathway, the LF cells were treated with 5 μM MSAB (#HY-120697, MedChemExpress, USA) to promote the degradation of β-catenin protein.

### CRISPR/Cas9 system and miR-335-3p mimics transfection

Two single guide RNAs targeting SERPINE2 locus were cloned into the U6-sgRNA-EF1a-Cas9-FLAG-CMV-EGFP-P2A-puro plasmid by GeneChem (Shanghai, China). The details of single guide RNA sequences were as follows: sgRNA-1: 5′-TACGCCGTATCTCATCACCA-3′ and sgRNA-2: 5′-TCAATCAGATTGTGAAGTCG-3′. In brief, the LF cells were transfected by the plasmids or miR-335-3p mimics with the assistance of Lipofectamine 3000 (L3000075) following the manufacturer’s instructions. The sequences of miR-335-3p mimics were listed in Supplementary Table 2.

### Lentivirus infection

To overexpress the SERPINE2 in LF cells, we used the lentivirus pSLenti-EF1-EGFP-P2A-Puro-CMV-SERPINE2-3xFLAG-WPRE to infect LF cells (OBiO Technology, China). In details, the LF cells were plated at 1.2 × 10^5^ cells/well in six-well plates containing serum-free culture medium with polybrene (2 µg/ml) at 37 °C in a 5% CO_2_ atmosphere. Then, LF cells were transduced with control or SERPINE2 lentivirus at a multiplicity of infection of 40 when reaching the 60–70% confluence. Six hours later, the cells were transferred to serum-containing medium and incubated for additional 2 days. The transduction efficiency was evaluated using the western blot to detect the protein level of SERPINE2.

### RNA immunoprecipitation (RIP) analysis

The RIP experiment was conducted with the PureBinding®RNA Immunoprecipitation Kit (#P0101, Geneseed, China) with anti-AGO2 (#2897, Cell Signaling Technology, USA), following the manufacturer’s instructions. The IgG (#3900, Cell Signaling Technology, USA) served as a negative control. The AGO2 antibody was recovered with the protein A/G beads. Co-precipitated SERPINE2 and miR-335-3p levels were assessed by qRT–PCR analysis.

### Western blot

Samples were lysed by a radioimmunoprecipitation assay buffer (#R0010, Solarbio, China) and their protein concentration were determined by BCA Protein Assay Kit (#PC0022, Solarbio, China). After centrifugation, the lysates were mixed with the loading buffer (#P1040, Solarbio, China) for western blot and then the mixture was heated for 15 min at 99 °C. Then, a total of 20 µg protein was added into the hole of 12% Bis–Tris gel (#LK306, Epizyme, China) and transferred to polyvinylidene fluoride (PVDF) membranes (#88518, Millipore, USA). The membranes were blocked for 1 h at room temperature with 5% non-fat dried milk in TBST, and then incubated for 2 h at room temperature with primary antibody, and for 1 h at room temperature with horseradish peroxidase (HRP)-conjugated secondary antibody (#SE134, #SE131, Solarbio, China). The detailed information for used antibody as follows: anti-COL3A1 (#ab184993, Abcam, UK), α-SMA (#ab7817, Abcam, UK), SERPINE2 (#11303-1-AP, Proteintech, USA), β-actin (#AF5003, Beyotime, China), and HRP-conjugated secondary antibody (#A0208, #A0216, Beyotime, China).

### RNA extraction and qRT-PCR assay

Total cellular RNA extraction was performed with the TRIzol method. The first-strand cDNA was obtained from total RNA using the commercial kits (#AG11705, # AG11716, Accurate Biology, China) according to the instructions. The quantitative real-time polymerase chain reaction (qRT-PCR) was conducted with the SYBR Green Supermix (#AG11702, Accurate Biology, China) according to the instructions. GAPDH and U6 were applied as the internal control for the mRNA and miRNA, respectively. The details of primers used in this study were listed in Supplementary Table 2.

### Dual luciferase assay

The human embryonic kidney (HEK) 293T cells (#CL-0005, Procell, China) were applied for the luciferase activity analysis. Generally, the HEK 293T cells were plated on 96‐well plates and cultured to 50–60% confluence. The 3′-UTR of SERPINE2 containing putative binding sites for miR-335-3p were cloned into the vector. The wild-type pMIR-REPORT-SERPINE2-3′-UTR and mutant luciferase reporter pMIR-REPORT-SERPINE2-3′-UTR were synthesized by GenePharma (Suzhou, China). A 150 ng vector of SERPINE2 3′-UTR-WT and SERPINE2 3′-UTR -MUT and 60 nM of miR-335-3p and negative control (NC) were transfected. The luciferase activity was examined with Dual Luciferase Reporter Assay kit (#RG029S, Beyotime, China) following the manufacturer’s instructions 48 h after the transfection.

### Immunohistochemistry (IHC) assay

Human LF specimens were treated with 4% paraformaldehyde (#P1110, Solarbio, China) for 48 h, and then were embedded in paraffin. Then, we used the microtome (Leica, Germany) to cut the paraffin samples into sections that were 4-μm thick. Next, the sections were then deparaffinized and hydrated. Then, the sections underwent staining using the hematoxylin and eosin (H&E) staining kit (#G1120, Solarbio, China). The Elastic Van Gieson (EVG) staining was conducted by EVG kit (#G1597, Solarbio, China) following the guidelines. An Olympus BX63 microscope (Olympus, Japan) was applied to observe the stained cells.

### Immunofluorescence (IF) staining

The LF cells were fixed for 20 min using 4% paraformaldehyde at room temperature. The fixed cells were treated with Triton X-100 (#T8200, Solarbio, China) and then subjected to immunoblocking. The fixed cells were left to incubate overnight at 4 °C with the primary antibodies listed below: anti-β-catenin (#ab32572, Abcam, UK) and anti-SERPINE2 (#11303-1-AP, Proteintech, USA). Then, fluorescein isothiocyanate (FITC)-conjugated secondary antibodies (#ab6717, Abcam, UK) and Cy3-conjugated secondary antibody (#ab6939, Abcam, UK) were used for IF staining. The DAB kit (#ab64238, Abcam, UK) was used for IHC staining, and DAPI (#C0065, Solarbio, China) was used for staining the nuclei in IF staining.

### Statistical analysis

All statistical analyses were conducted using the GraphPad Prism 9.0 (La Jolla, USA) and all results were showed as the mean ± standard deviation (SD). The data normality was assessed with the Shapiro–Wilk test. For the comparisons of continuous variables between two groups, the unpaired two-tailed *t*-tests were used for normally distributed data, and Wilcoxon rank-sum test was used to compare group approaches for nonparametric data. For the comparisons across more than two groups, one-way analysis of variance (ANOVA) tests followed by Tukey’s honestly significant difference (HSD) multiple comparison tests were applied were used. The *P* < 0.05 was determined to be a significant difference (ns, not significant; *P < 0.05, **P < 0.01, ***P < 0.001, and ****P < 0.0001).

## Results

### Fibrosis of LF cells in patients with LFH

**Fig. 1:**
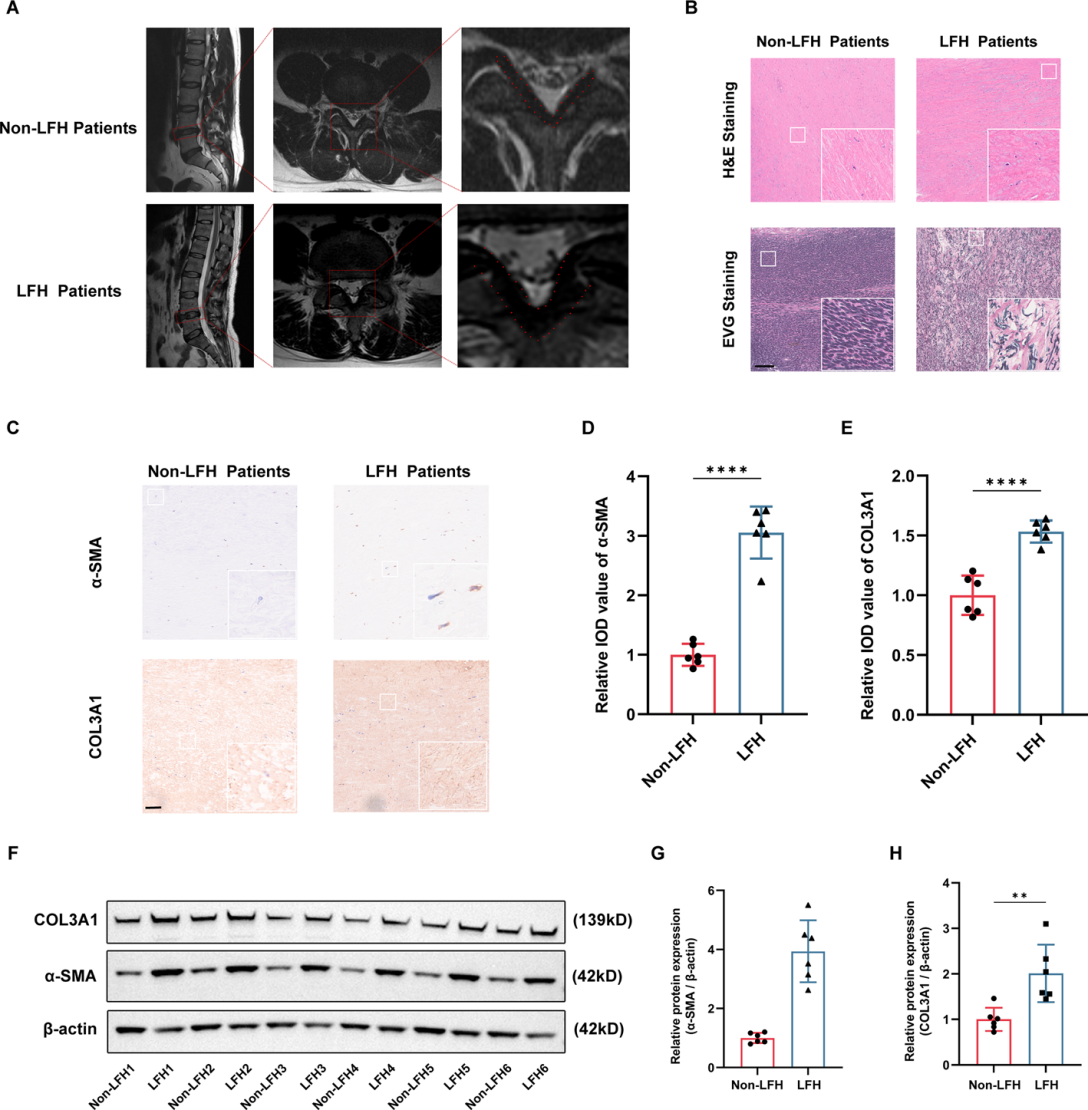
Fibrosis of LF cells is the main pathological characteristic of LFH. **A** The LF tissues in MRI. **B** The H&E staining and EVG staining of LF tissues. **C**–**H** The protein expression of fibrosis-related markers was evaluated using IHC and western blotting. Scale bar, 100 μm. *P < 0.05, **P < 0.01, ***P < 0.001, and ****P < 0.0001

The MRI results showed that the thickness of LF tissues in patients with LFH was significantly thicker than patients without LFH (Fig. 1A). Previous studies reported that the fibrosis of LF was the main pathologic change during the process of LFH [2]. In LFH tissues, the H&E staining presented the elastin fibers were irregularly arranged and ruptured in LFH tissues. On the contrary, there were lots of regularly arranged elastin fibers in non-HLF patients. The EVG staining showed that the elastic fibers were reduced and replaced with lots of collagen fibers (Fig. 1B). We used the IHC and western blotting to determine the expression of fibrosis-related markers (α-SMA and COL3A1), and the protein expression of fibrosis-related markers (α-SMA and COL3A1) were significantly elevated in LFH tissues compared to non-LFH tissues (Fig. 1C–H).

### MiR-335-3p was downregulated in the LFH

**Fig. 2:**
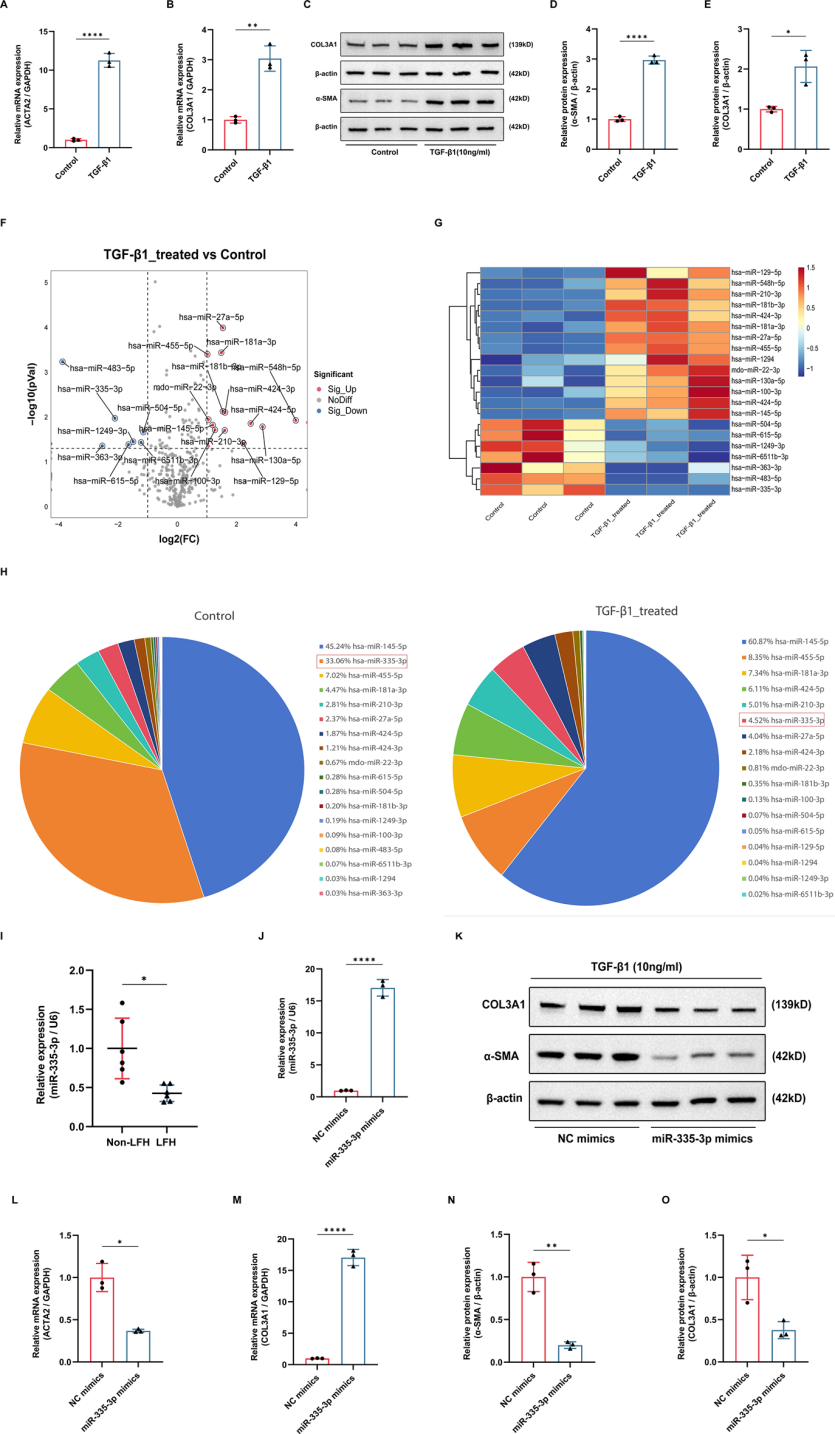
Overexpression of miR-335-3p inhibited the TGF-β1-induced fibrosis of LF cells. **A**–**E** The mRNA and protein expression of fibrosis-related markers after the TGF-β1 treatment. **F**, **G** The volcano plot and heat map of differently expressed miRNAs after the TGF-β1 treatment. **H** The abundance of miR-335-3p after the TGF-β1 treatment. **I** The expression of miR-335-3p in LF tissues was evaluated using the qRT-PCR. **J** The transfection efficiency of miR-335-3p mimics was checked using the qRT-PCR. **K**–**O** The mRNA and protein expression of fibrosis-related markers after the miR-335-3p overexpression using the qRT-PCR and western blot. *P < 0.05, **P < 0.01, and ****P < 0.0001

Epigenetic modification is proved to play important roles in spinal degenerative diseases [6, 21, 22]. In this study, we paid special attention to the epigenetic regulator miRNAs in the pathogenesis of LFH. TGF-β1 was considered as the most important profibrotic factor in many fibrotic diseases, including the LFH [5, 23]. Therefore, we used the TGF-β1 (10 ng/ml) to treat LF cells to construct the fibrosis model of LF cells. The qRT-PCR and western blotting results showed the fibrosis-related markers were obviously increased in LF cells treated with TGF-β1 compared with control LF cells in mRNA level (ACTA2 and COL3A1) (Fig. 2A, B) and protein level (α-SMA and COL3A1) (Fig. 2C–E), which demonstrated that we have successfully constructed the cell model of LFH. To explore the role of miRNAs in the pathogenesis of LFH, we conducted the miRNA sequencing analysis of LF cells treated with TGF-β1. A total of 21 miRNAs were aberrantly expressed during the fibrotic process of LF cells (fold change > 2, *P* < 0.05), including 7 downregulated miRNAs and 14 upregulated miRNAs (Fig. 2F, G). The downregulated miRNAs caught our attention for their protective roles in the pathogenesis of LFH. Among the seven downregulated miRNAs, we specially selected the miR-335-3p for further analysis because its’ abundance was also significantly reduced during the fibrosis of LF cells (Fig. 2H). Then, qRT-PCR confirmed that miR-335-3p was downregulated in LFH tissues compared to non-LFH tissues (Fig. 2I). All findings demonstrated that miR-335-3p might play an important role in the LFH.

### Overexpression of miR-335-3p inhibited the TGF-β1-induced fibrosis of LF cells

To further explore the role of miR-335-3p in LFH, we transfected the LF cells with miR-335-3p mimics to increase the expression level of miR-335-3p (Fig. 2J). The western blotting results presented that the transfection of miR-335-3p mimics could obviously reduce the protein level of fibrosis-related markers (α-SMA and COL3A1) induced by the TGF-β1 (Fig. 2K–M). The qRT-PCR results showed that overexpression of miR-335-3p could significantly decrease the mRNA level of fibrosis-related markers (ACTA2 and COL3A1) induced by TGF-β1 (Fig. 2N, O). Therefore, our findings showed that overexpression of miR-335-3p could relieve the fibrosis of LF cells induced by TGF-β1.

### MiR-335-3p repressed SERPINE2 expression by targeting the 3′-UTR of SERPINE2

**Fig. 3:**
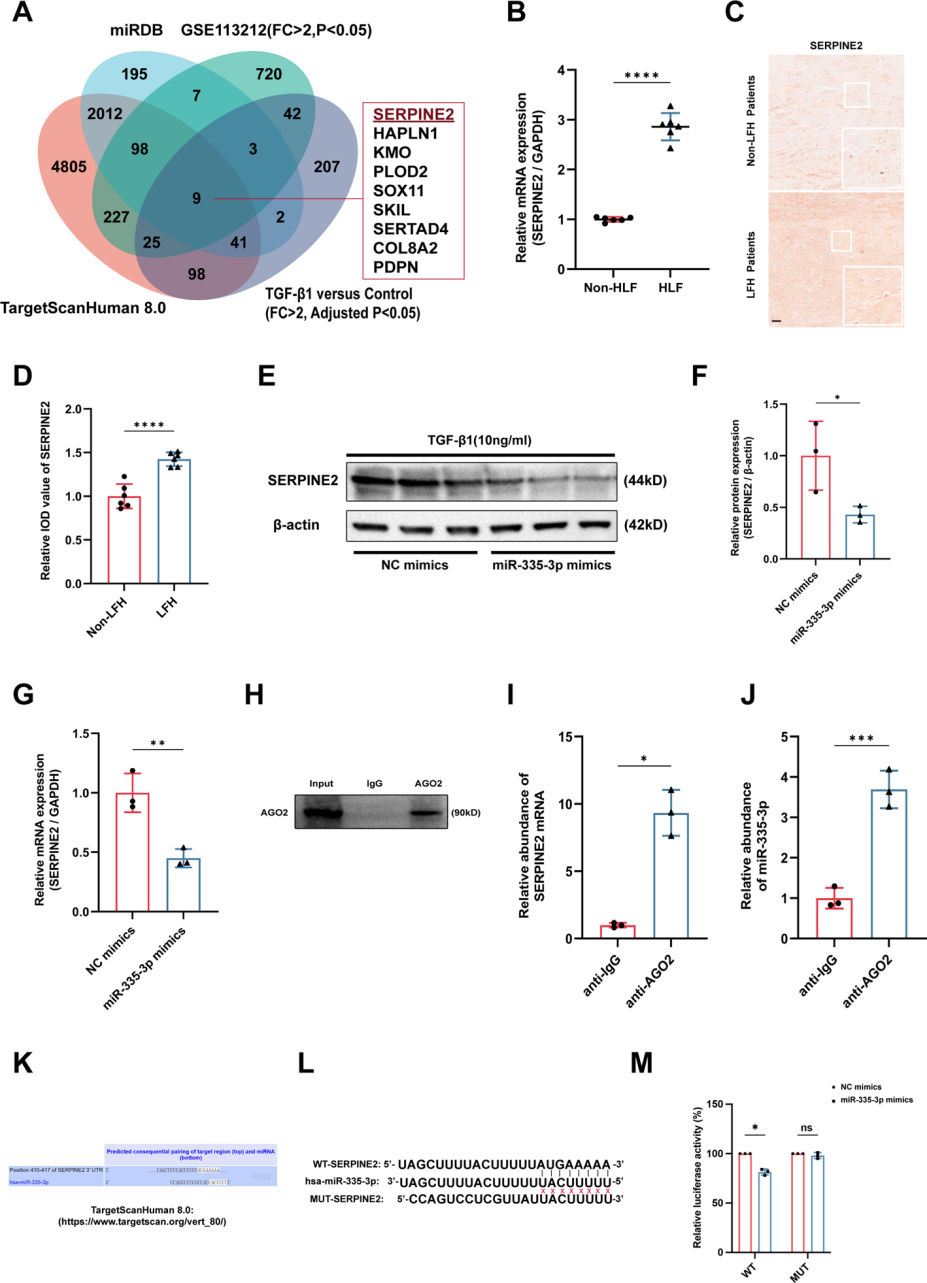
MiR-335-3p repressed SERPINE2 expression by targeting the 3′-UTR of SERPINE2. **A** The bioinformatic analysis showed SERPINE2 was the potential target of miR-335-3p. **B**–**D** The mRNA and protein expression of SERPINE2 in LF tissues were evaluated through the qRT-PCR and IHC. **E**–**G** The mRNA and protein expression of SERPINE2 after the miR-335-3p overexpression were assessed using the qRT-PCR and western blot. **H**–**J** The relationship among miR-335-3p, SERPINE2, and AGO2 was assessed based on RIP analysis. **K** The potential binding sites between miR-335-3p and SERPINE2 were predicted using the TargetScanHuman 8.0 database. **L**, **M** The binding sites between miR-335-3p and SERPINE2 were identified using the dual luciferase assay. Scale bar, 100 μm. ns, not significant, *P < 0.05, **P < 0.01, ***P < 0.001, and ****P < 0.0001

The most common biological function of miRNAs in the epigenetic modification is to promote the degradation or suppress the translation through binding the 3′-UTR of targeted genes [13]. To explore the potential downstream targets of miR-335-3p in LFH, we used the TargetScanHuman 8.0 (https://www.targetscan.org/vert_80/) and miRDB database (https://mirdb.org/) to predict the potential targets of miR-335-3p based on the bioinformatic analysis. Then, the targeted mRNAs of miR-335-3p predicted by bioinformatic analysis were further intersected with upregulated mRNAs (fold change > 2, *P* < 0.05) in HLF tissues (GSE113212) and (fold change > 2, adjusted *P* < 0.05) TGF-β1-treated LF cells (Supplementary Table 3). At last, nine potential targets of miR-335-3p were obtained. In particular, SERPINE2 caught our attention because it has been reported to be involved in other fibrotic diseases [17, 19] (Fig. 3A). The qRT-PCR and IHC showed that mRNA level and protein level of SERPINE2 were both significantly increased in HLF tissues compared with non-HLF tissues (Fig. 3B–D). To further explore the regulator role of miR-335-3p on the SERPINE2, we transfected the LF cells with miR-335-3p mimics, and our results showed that protein and mRNA levels of SERPINE2 were both obviously decreased after the transfection of miR-335-3p mimics (Fig. 3E–G). The classical mechanisms for miRNAs degrading mRNA and inhibiting translation is directly binding to their targets in an AGO2-dependent manner [12, 13]; therefore, we conducted the anti-AGO2 RNA immunoprecipitation (RIP) in LF cells. Our results showed that miR-335-3p and SERPINE2 pulled down by anti-AGO2 antibody were both significantly enriched compared with negative anti-IgG antibody (Fig. 3H–J). To confirm the binding sites between miR-335-3p and SERPINE2, based on the bioinformatic analysis of TargetScanHuman 8.0 (https://www.targetscan.org/vert_80/) (Fig. 3K), we constructed a luciferase reporter vector with the wild-type (WT) or mutant (MUT) SERPINE2 3′-UTR containing the putative miR-335-3p target site, and our data indicated that miR-335-3p overexpression significantly decreased the luciferase activity of the reporter containing the WT 3′-UTR of SERPINE2 compared with mimic control, but there was no obvious change in the luciferase activity in the MUT 3′-UTR of SERPINE2 group (Fig. 3L, M).

### Knockdown of SERPINE2 inhibited the TGF-β1-induced fibrosis of LF cells

**Fig. 4:**
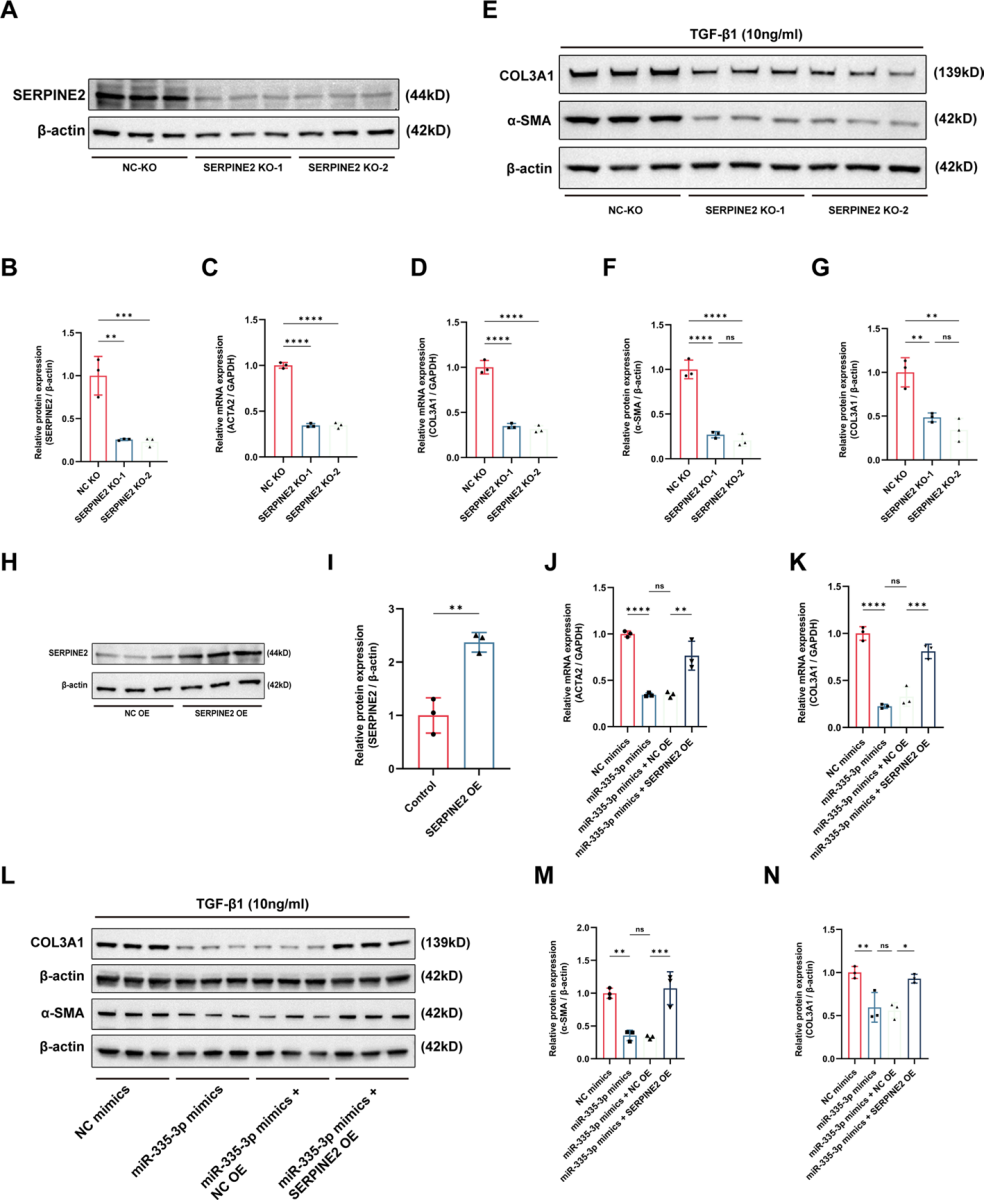
Knockdown of SERPINE2 inhibited the TGF-β1-induced fibrosis of LF cells. **A**, **B** The efficacy of SERPINE2 knockdown was assessed using the western blot. **C**–**G** The mRNA and protein expression of fibrosis-related markers after the SERPINE2 knockdown was evaluated using the qRT-PCR and western blot. **H**, **I** The protein expression of SERPINE2 after the SERPINE2 overexpression was evaluated using the western blot. **J**–**N** The mRNA and protein expression of fibrosis-related markers in the rescue experiments between miR-335-3p and SERPINE2. ns, not significant; *P < 0.05, **P < 0.01, ***P < 0.001, and ****P < 0.0001

To determine the role of SERPINE2 in the fibrosis of LF cells, we used the CRISPR/Cas9 technology to knock out SERPINE2 in LF cells. The protein level of SERPINE2 was significantly decreased after the transfection of two vectors carrying the CRISPR-Cas9 targeting the SERPINE2 (Fig. 4A, B). The qRT-PCR results showed that increased mRNA level of fibrosis-related markers (ACTA2 and COL3A1) induced by the TGF-β1 could be significantly inhibited by the reduction of SERPINE2 (Fig. 4C, D). Similarly, the protein level of fibrosis-related markers (α-SMA and COL3A1) caused by the TGF-β1 were decreased after the knockdown of SERPINE2 during the fibrosis of LF cells (Fig. 4E–G). In overall, knockdown of SERPINE2 could inhibit the TGF-β1 induced fibrosis of LF cells.

The rescue experiments were conducted to further determine the effect of miR-335-3p/SERPINE2 axis on the fibrosis of LF cells. We used the lentiviral vector containing SERPINE2 to infect the LF cells to overexpress the SERPINE2 in LF cells, and the protein level of SERPINE2 was significantly increased after the infection (Fig. 4H, I). The qRT-PCR results presented that the increased mRNA level of fibrosis-related markers (ACTA2 and COL3A1) induced by TGF-β1 could be reduced by the transfection of miR-335-3p mimics, but the reduction was abolished by the overexpression of SERPINE2 (Fig. 4J, K). Similarly, the protein level of fibrosis-related markers (α-SMA and COL3A1) were significantly reduced by the miR-335-3p overexpression, but the reduction was rescued by the overexpression of SERPINE2 (Fig. 4L–N). Overall, our results confirmed the important role of miR-335-3p/SERPINE2 axis in the fibrosis of LF cells.

### Overexpression of SERPINE2 promoted the TGF-β1-induced fibrosis of LF cells through the activation of β-catenin signaling pathway

**Fig. 5:**
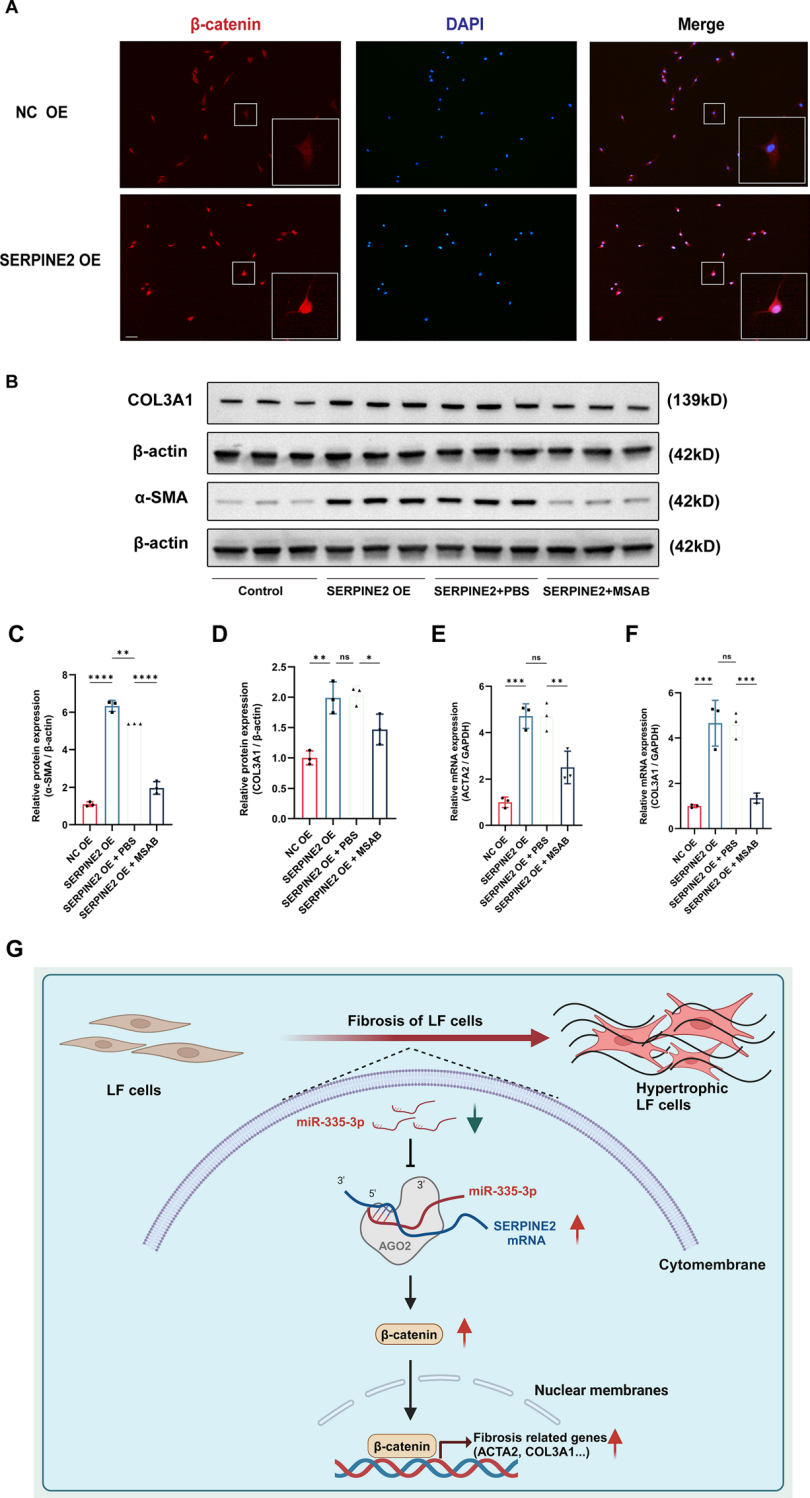
Overexpression of SERPINE2 promoted the TGF-β1-induced fibrosis of LF cells through the activation of β-catenin signaling pathway. **A** The activation of β-catenin signaling pathway was evaluated using the immunofluorescence after the SERPINE2 overexpression. **B**–**F** The mRNA and protein expression of fibrosis-related markers in the rescue experiments between SERPINE2 and β-catenin signaling pathway. **G** Epigenetic modification regulated the ligamentum flavum hypertrophy through miR-335-3p/SERPINE2/β-catenin signaling pathway. Scale bar, 100 μm. ns, not significant, *P < 0.05, **P < 0.01, ***P < 0.001, and ****P < 0.0001

Activation of β-catenin signaling pathway has been confirmed to play vital roles in the fibrotic diseases [24, 25]. SERPINE2 was previously reported to promote the myocardial fibrosis by activating the ERK1/2 and β-catenin signaling pathways [17]. Therefore, we speculated that the SERPINE2 promoted the fibrosis of LF cells via activating the β-catenin signaling pathway. To determine the role of SERPINE2 in the regulation of β-catenin signaling pathway, we infected the LF cells with lentiviral vector containing SERPINE2 to overexpress the SERPINE2 expression. The immunofluorescence results showed the nuclear translocation of β-catenin was increased in LF cells after the overexpression of SERPINE2, which indicated that SERPINE could activate the β-catenin signaling pathway (Fig. 5A). To confirm the SERPINE2/β-catenin pathway in the fibrosis of LF cells, we used the lentiviral vector containing SERPINE2 and 5 μM MSAB (a β-catenin signaling inhibitor, HY120697, MCE, USA) to treat the LF cells, and western blot results showed that the increased protein level of fibrosis-related markers (α-SMA and COL3A1) caused by SERPINE2 overexpression could be partly abolished by the addition of MSAB (Fig. 5B–D). Similarly, the mRNA level of fibrosis-related markers (ACTA2 and COL3A1) were obviously increased in LF cells after the SERPINE2 overexpression, but this tendency was reduced by the addition of MSAB (Fig. 5E, F). Therefore, our findings indicated that SERPINE2 promoted the fibrosis of LF cells through the activation of β-catenin signaling pathway to promote the transcription of fibrosis-related genes (ACTA2 and COL3A1).

In summary, our findings presented that the miR-335-3p was decreased during the fibrosis of LF cells, which further promoted the SERPINE2 expression, and then activated the β-catenin signaling pathway to stimulate the transcription of fibrosis-related genes (ACTA2 and COL3A1) to promote the fibrosis of LF cells (Fig. 5G).

## Discussion

LSS has become the leading cause of low back pain, which brings heavy economic burden to individuals and society [1]. One important reason for LSS is the LFH, in which the LFH tissues compress the spinal cord and nerves, leading to neurological disorders [1]. Previous studies showed the main pathological change of LFH is the fibrosis of LF cells, with the characteristics of myofibroblastic transformation of LF cells and deposition of collagens [2, 5]. Several factors are reported to be associated with the pathogenesis of LFH, such as obesity, aging, and mechanical stress [26]. However, the exact molecular mechanism of LFH remains obscure, to date.

In our study, similar to previous studies [2, 3, 5, 23], we demonstrated that fibrosis of LF cells is the main pathological feature of LFH tissues, which remind us that the inhibition of the fibrosis of LF cells is the key point in the therapeutic of LFH. miRNAs have been proven to play important roles in many human diseases [8, 27, 28], including LFH [29–31]. The miR-221 was significantly decreased in hypertrophic LF tissues and overexpression of miR-221 could relieve the fibrosis of LF cells by targeting the TIMP-2 [29]. The miR-10396b-3p was reduced in hypertrophic LF tissues than normal LF tissues, and overexpression of miR-10396b-3p could inhibit the fibrosis of LF cells through regulating the IL-11 [31]. The miR-21 was reported to promote the fibrosis of LF cells by increasing the expression of fibrosis-related genes and IL-6 [30]. MiR-335-3p are demonstrated to be associated with the pathogenesis of several human diseases [14, 32, 33]. Fan et al. reported that NF-κB transcriptional regulation increased the miR-335-3p level, which further promoted the induction of pulmonary arterial hypertension by targeting the APJ [14]. Jia et al. found that transcriptional factor FOXM1 could activate the miR-335-3p to maintain the self-renewal of neural stem cells via targeting the FMR1 to suppress the p53 signaling pathway [33]. Ju et al. study indicated that miR-335-3p could improve the type II diabetes mellitus by regulating macrophage polarization through targeting the IGF-1 [32]. However, few studies report the vital role of miR-335-3p in fibrotic diseases. In this study, based on the miRNA sequencing results, we found that miR-335-3p was significantly downregulated during the fibrosis of LF cells. The further in vitro experiments showed that overexpression of miR-335-3p could obviously inhibit the fibrosis of LF cells. Therefore, our results indicated that miR-335-3p is a promising therapeutic target for LFH. To our knowledge, our study is first to report the anti-fibrotic role of miR-335-3p in LFH.

Epigenetic modification is involved in the occurrence and progression of spinal diseases [6]. miRNAs, as a crucial epigenetic factor, exert their biological functions through binding to the complementary sequences in the 3′-UTR of targeted genes to degrade the mRNA or inhibit the translation process of targeted genes [12, 13]. In our study, we predicted that SERPINE2 is the potential targeted gene of miR-335-3p based on the bioinformatic analysis and sequencing analysis. Then, we conducted the dual-luciferase reporter assay and RIP analysis demonstrated that miR-335-3p regulated the SERPINE2 expression through binding to its’ seed region of 3′-UTR to promote the degradation of SERPINE2 mRNA. Moreover, the rescue experiments showed that antifibrotic effects of miR-335-3p overexpression was partly abolished by the overexpression of SERPINE2. Therefore, our study demonstrated that miR-335-3p regulated the fibrosis of LF cells through the epigenetic modification by targeting the SERPINE2.

SERPINE2, as a famous protease inhibitor, has been reported to promote the fibrosis in fibrotic diseases [17, 18]. SERPINE2 was reported to be translocated into cardiac fibroblasts through the endocytosis, and then activated the ERK1/2 and β-catenin signaling pathways to promote the collagen production in cardiac fibrosis [17, 34]. Besides, SERPINE2 was promoted by the MEK1/2-ERK1/2 pathway via the transcription factors Elk1 during the cardiac fibrosis [18]. β-catenin signaling pathway has been proved to facilitate the fibrosis by promoting the transcription of fibrotic genes in fibrotic diseases, including the LFH [35, 36]. In our study, similar to the previous research [17], we found that overexpression of SERPINE2 could activate the β-catenin signaling pathway during the fibrosis of LF cells. The profibrotic effects caused by the SERPINE2 overexpression was significantly decreased with the addition of a β-catenin inhibitor MSAB. Overall, our findings showed that epigenetic modification regulated the fibrosis of LF cells through the miR-335-3p/SERPINE2/β-catenin signaling pathway.

Some limitations should be considered when interrupting our results. First, although several animal models have been established to stimulate the process of LF hypertrophy, none of them could successfully induce the clinical symptoms relevant to LF hypertrophy, such as intermittent claudication and low back pain [37, 38]. Therefore, the in vivo experiments are not conducted in this study. Further studies should be conducted to develop better animal LFH models. Second, we discovered that SERPINE2 could promote the fibrosis of LF cells through the activation of β-catenin signaling pathway. However, the detailed mechanism about the interaction between SERPINE2 and β-catenin signaling pathway remains unclear, which needs further investigation.

## Conclusions

Our research proposed a mechanism in the fibrosis of LF cells mediated by epigenetic modification, manifesting that targeting the miR-335-3p/SERPINE2/β-catenin signaling pathway could alleviate the LFH and provide a promising epigenetic therapeutic strategy for LFH treatment.

## Supplementary Information

Supplementary Material 1.

## Data Availability

The data generated during the current study are available from the corresponding author upon reasonable request.

## Abbreviations

LSS: Lumbar spinal stenosis
LFH: Ligamentum flavum hypertrophy
LF: Ligamentum flavum
miRNA: MicroRNA
UTR: Untranslated region
SERPINE2: Serpin family E member 2
MRI: Magnetic resonance imaging
RIP: RNA immunoprecipitation
HEK: Human embryonic kidney
H&E staining: Hematoxylin and eosin staining
EVG staining: Elastic-Van Gieson staining
IHC: Immunohistochemistry
IF: Immunofluorescence
