# Supplementary material for: Epigenetic modification regulates the ligamentum flavum hypertrophy through miR-335-3p/SERPINE2/β-catenin signaling pathway
Source: Cell Mol Biol Lett. 2025 Jan 3;30:1. doi: 10.1186/s11658-024-00660-z (PMC11699792; doi:10.1186/s11658-024-00660-z)
Supplement: Supplementary file 1 — Supplementary Material 1. [file 11658_2024_660_MOESM1_ESM.docx]

**Supplementary Table 1 Clinical information of included patients in this study.**

| **ID** | **Gender** | **Age, year** | **Diagnosis** | **Application** |
| --- | --- | --- | --- | --- |
| 1 | Male | 35 | LDH | Cell extraction, RNA level evaluation of miR-335-3p and SERPINE2, H&E staining, and EVG staining. |
| 2 | Female | 42 | LDH | Cell extraction, RNA level evaluation of miR-335-3p and SERPINE2, H&E staining, and EVG staining. |
| 3 | Male | 29 | LDH | Cell extraction, RNA level evaluation of miR-335-3p and SERPINE2, H&E staining, and EVG staining. |
| 4 | Male | 47 | LDH | Cell extraction, RNA level evaluation of miR-335-3p and SERPINE2, H&E staining, and EVG staining. |
| 5 | Male | 51 | LDH | Cell extraction, RNA level evaluation of miR-335-3p and SERPINE2, H&E staining, and EVG staining. |
| 6 | Female | 39 | LDH | Cell extraction, RNA level evaluation of miR-335-3p and SERPINE2, H&E staining, and EVG staining. |
| 7 | Female | 47 | LDH | Cell extraction, Protein level evaluation of α-SMA, COL3A1, and SERPINE2 |
| 8 | Male | 37 | LDH | Cell extraction, Protein level evaluation of α-SMA, COL3A1, and SERPINE2 |
| 9 | Male | 48 | LDH | Cell extraction, Protein level evaluation of α-SMA, COL3A1, and SERPINE2 |
| 10 | Female | 42 | LDH | Cell extraction, Protein level evaluation of α-SMA, COL3A1, and SERPINE2 |
| 11 | Female | 23 | LDH | Cell extraction, Protein level evaluation of α-SMA, COL3A1, and SERPINE2 |
| 12 | Male | 19 | LDH | Cell extraction, Protein level evaluation of α-SMA, COL3A1, and SERPINE2 |
| 13 | Male | 43 | LFH | RNA level evaluation of miR-335-3p and SERPINE2, H&E staining, and EVG staining. |
| 14 | Male | 56 | LFH | RNA level evaluation of miR-335-3p and SERPINE2, H&E staining, and EVG staining. |
| 15 | Female | 65 | LFH | RNA level evaluation of miR-335-3p and SERPINE2, H&E staining, and EVG staining. |
| 16 | Male | 68 | LFH | RNA level evaluation of miR-335-3p and SERPINE2, H&E staining, and EVG staining. |
| 17 | Male | 51 | LFH | RNA level evaluation of miR-335-3p and SERPINE2, H&E staining, and EVG staining. |
| 18 | Female | 76 | LFH | RNA level evaluation of miR-335-3p and SERPINE2, H&E staining, and EVG staining. |
| 19 | Female | 61 | LFH | Protein level evaluation of α-SMA, COL3A1, and SERPINE2 |
| 20 | Male | 43 | LFH | Protein level evaluation of α-SMA, COL3A1, and SERPINE2 |
| 21 | Male | 59 | LFH | Protein level evaluation of α-SMA, COL3A1, and SERPINE2 |
| 22 | Female | 63 | LFH | Protein level evaluation of α-SMA, COL3A1, and SERPINE2 |
| 23 | Female | 72 | LFH | Protein level evaluation of α-SMA, COL3A1, and SERPINE2 |
| 24 | Male | 48 | LFH | Protein level evaluation of α-SMA, COL3A1, and SERPINE2 |

LDH, lumbar disc herniation; LFH, ligamentum flavum hypertrophy; EVG, Elastica van Gieson.

**Supplementary Table 2. Details of primers and miR-335-3p mimics.**

| **Primers** |  |
| --- | --- |
| Has-miR-335-3p | 5’- GGGCGTTTTTCATTATTGCTCCTG-3’ |
| COL3A1-Forward | 5’- ATATCGAACACGCAAGGCTGTG-3’ |
| COL3A1-Reverse | 5’- GCCAACGTCCACACCAAATTC -3’ |
| ACTA2-Forward | 5’- TCACCCACAATGTCCCCATCTATG -3’ |
| ACTA2-Reverse | 5’- ATCTCACGCTCAGCAGTAGTAACG -3’ |
| SERPINE2-Forward | 5’- AGCCTCTGCCTGTGATTCCATC -3’ |
| SERPINE2-Reverse | 5’- ACCCTTGAAATACACTGCGTTGAC -3’ |
| GAPDH-Forward | 5’- CAGGAGGCATTGCTGATGAT -3’ |
| GAPDH-Reverse | 5’- GAAGGCTGGGGCTCATTT -3’ |
| U6-Forward | 5’- CTCGCTTCGGCAGCACA -3’ |
| U6-Reverse | 5’- AACGCTTCACGAATTTGCGT- 3’ |
| **Has-miR-335-3p mimics** | 5’- TTTTTCATTATTGCTCCTGACC -3’ |

**Supplementary Table 3. Upregulated genes in TGF-β1 treated LF cells.**

| **Gene name** | **Fold change** | **Adjusted P value** |
| --- | --- | --- |
| CACNA1H | 11.06 | 0 |
| TPM1 | 2.97 | 0 |
| SLC15A1 | 29.72 | 0 |
| LIF | 11.40 | 0 |
| LDLRAD4 | 18.15 | 0 |
| FSTL3 | 3.09 | 0 |
| PLCB4 | 3.20 | 0 |
| COL8A2 | 4.81 | 0 |
| TGFBI | 8.01 | 0 |
| RFLNB | 5.96 | 0 |
| COL4A1 | 3.25 | 0 |
| MAMDC2 | 12.22 | 0 |
| SYNE1 | 2.52 | 0 |
| COL4A2 | 2.56 | 0 |
| APCDD1L | 4.47 | 0 |
| NTF4;CGB8;CGB7 | 8.86 | 0 |
| CNN1 | 7.61 | 0 |
| IVNS1ABP | 4.32 | 0 |
| TNS1 | 3.08 | 0 |
| MYH10 | 2.27 | 0 |
| TGM2 | 5.20 | 0 |
| DGKI | 4.92 | 0 |
| HAPLN1 | 4.45 | 0 |
| DSP | 3.85 | 0 |
| NUAK1 | 3.95 | 0 |
| FOXS1 | 4.60 | 0 |
| PPME1 | 9.94 | 0 |
| WNT5A | 2.30 | 0 |
| P4HA3 | 10.88 | 0 |
| COL7A1 | 5.33 | 0 |
| ANXA8L1 | 4.01 | 0 |
| PRPS1 | 4.32 | 0 |
| CLEC18B | 24.44 | 0 |
| INHBA | 4.13 | 0 |
| PMEPA1 | 10.96 | 0 |
| IER3 | 7.69 | 0 |
| CDKN2B | 8.09 | 0 |
| XYLT1 | 5.63 | 0 |
| SH3PXD2A | 4.49 | 0 |
| ADAM19 | 5.11 | 0 |
| CLEC18C | 24.98 | 0 |
| GPAM | 3.99 | 0.00 |
| MFAP3;GALNT10 | 2.20 | 0.00 |
| ADAM12 | 2.84 | 0.00 |
| MBOAT2 | 3.57 | 0.00 |
| DACT1 | 2.29 | 0.00 |
| LTBP2 | 2.50 | 0.00 |
| FAP | 2.73 | 0.00 |
| CELSR1 | 2.66 | 0.00 |
| DYSF | 10.06 | 0.00 |
| TSPAN2 | 11.94 | 0.00 |
| BHLHE40 | 3.70 | 0.00 |
| PKNOX2 | 2.58 | 0.00 |
| ANKRD1 | 4.97 | 0.00 |
| KIF26B | 3.38 | 0.00 |
| CRLF1 | 6.77 | 0.00 |
| KRT18 | 2.75 | 0.00 |
| NT5DC2 | 2.48 | 0.00 |
| MICAL2 | 2.51 | 0.00 |
| SRPX2 | 5.06 | 0.00 |
| CHST11 | 4.83 | 0.00 |
| PLXDC2 | 2.41 | 0.00 |
| STK38L | 2.56 | 0.00 |
| ADAMTS6 | 2.88 | 0.00 |
| PDGFC | 2.27 | 0.00 |
| SDC1 | 4.65 | 0.00 |
| SGK1 | 5.89 | 0.00 |
| SLC38A5 | 2.55 | 0.00 |
| WNT5B | 3.40 | 0.00 |
| BMP6 | 14.53 | 0.00 |
| COL5A1 | 2.25 | 0.00 |
| IGFBP3 | 2.53 | 0.00 |
| LRRC15 | 6.26 | 0.00 |
| IGFBP5 | 2.71 | 0.00 |
| APBB2;ENSG00000289761 | 2.45 | 0.00 |
| KIAA0040 | 16.18 | 0.00 |
| GXYLT2;PPP4R2 | 2.86 | 0.00 |
| DCLK2 | 2.23 | 0.00 |
| CTPS1 | 3.35 | 0.00 |
| NRP2 | 2.62 | 0.00 |
| SERPINE2 | 2.27 | 0.00 |
| GDF6 | 3.23 | 0.00 |
| FRMD6 | 2.07 | 0.00 |
| MMP16 | 2.47 | 0.00 |
| KRT86;KRT7 | 2.09 | 0.00 |
| PTPRE | 3.27 | 0.00 |
| SKIL | 2.46 | 0.00 |
| SERTAD4 | 10.32 | 0.00 |
| TGFB1 | 2.18 | 0.00 |
| CSRP1 | 2.05 | 0.00 |
| ADAMTS4 | 5.03 | 0.00 |
| SLC2A1 | 2.59 | 0.00 |
| POSTN | 2.30 | 0.00 |
| PRICKLE2 | 2.49 | 0.00 |
| VCAN | 2.40 | 0.00 |
| PLOD2 | 2.24 | 0.00 |
| PI16 | 3.79 | 0.00 |
| NPR3 | 2.14 | 0.00 |
| WSB2 | 2.24 | 0.00 |
| INHBE;GLI1 | 3.16 | 0.00 |
| PXDC1 | 2.19 | 0.00 |
| SPECC1 | 2.55 | 0.00 |
| ACTA2 | 2.11 | 0.00 |
| NRG1;ENSG00000286131 | 2.38 | 0.00 |
| SYNDIG1 | 2.95 | 0.00 |
| C5orf46 | 9.56 | 0.00 |
| VDR | 2.30 | 0.00 |
| ATP10A | 2.12 | 0.00 |
| ENC1 | 3.11 | 0.00 |
| WNK4 | 10.13 | 0.00 |
| RHOB | 2.02 | 0.00 |
| KCNG1 | 2.77 | 0.00 |
| PCED1B | 3.17 | 0.00 |
| PRR5L | 3.10 | 0.00 |
| PCDH1 | 3.39 | 0.00 |
| SNAI1 | 3.96 | 0.00 |
| FN1 | 2.13 | 0.00 |
| LTBP4 | 2.22 | 0.00 |
| ZNF281 | 2.26 | 0.00 |
| SMAD7 | 2.02 | 0.00 |
| HSPA13 | 2.22 | 0.00 |
| SOX4 | 2.15 | 0.00 |
| C2CD3 | 2.25 | 0.00 |
| NOL4L | 2.04 | 0.00 |
| PDGFA | 2.00 | 0.00 |
| TCF4 | 2.01 | 0.00 |
| CSRNP1 | 2.86 | 0.00 |
| TNFRSF10D | 2.55 | 0.00 |
| TENM2 | 2.20 | 0.00 |
| PGF | 8.84 | 0.00 |
| TENM3 | 2.22 | 0.00 |
| BDNF | 2.64 | 0.00 |
| TENM4 | 3.37 | 0.00 |
| TIPARP | 2.32 | 0.00 |
| GDNF | 5.73 | 0.00 |
| ETV5;DGKG | 2.27 | 0.00 |
| LGMN | 2.16 | 0.00 |
| SLC19A2 | 4.52 | 0.00 |
| IFFO2 | 2.31 | 0.00 |
| EXTL1 | 11.26 | 0.00 |
| DCBLD1 | 2.49 | 0.00 |
| MEX3B | 2.42 | 0.00 |
| CDH2 | 6.31 | 0.00 |
| GLIPR2 | 2.03 | 0.00 |
| TMPO | 2.28 | 0.00 |
| ABLIM3 | 2.24 | 0.00 |
| FBXO32 | 2.70 | 0.00 |
| NOX4 | 18.17 | 0.00 |
| SYT16 | 7.56 | 0.00 |
| SERPINE1 | 5.51 | 0.00 |
| FIBIN | 2.33 | 0.00 |
| IL6 | 3.69 | 0.00 |
| MARCHF4 | 2.24 | 0.00 |
| PGM2L1 | 2.94 | 0.00 |
| HDGFL3 | 2.13 | 0.00 |
| LIMK2 | 2.29 | 0.00 |
| MSMO1 | 2.27 | 0.00 |
| MDFI | 7.64 | 0.00 |
| PODNL1 | 2.46 | 0.00 |
| PLXNA4 | 2.97 | 0.00 |
| DMRTA1 | 31.18 | 0.00 |
| DNM1 | 2.62 | 0.00 |
| P2RY6 | 3.21 | 0.00 |
| MYOCD | 3.81 | 0.00 |
| SLC22A3 | 31.76 | 0.00 |
| FNDC1 | 2.22 | 0.00 |
| NALCN | 2.43 | 0.00 |
| MFAP3L | 4.08 | 0.00 |
| ALPK3 | 3.17 | 0.00 |
| CLEC18A | 27.94 | 0.00 |
| TBX3 | 2.79 | 0.00 |
| SORCS2 | 2.52 | 0.00 |
| HOXB9 | 3.67 | 0.00 |
| PTGS1 | 14.31 | 0.00 |
| PSD4 | 3.17 | 0.00 |
| PLAUR | 2.33 | 0.00 |
| ENSG00000284299;KCNH1 | 2.27 | 0.00 |
| ATP2A3 | 2.24 | 0.00 |
| PDPN | 2.08 | 0.00 |
| LHB;ENSG00000268655 | 8.36 | 0.00 |
| PTPRN | 5.84 | 0.00 |
| AMIGO2 | 20.27 | 0.00 |
| PNP | 2.12 | 0.00 |
| IL21R | 4.98 | 0.00 |
| TRIM62 | 2.14 | 0.00 |
| NCF2 | 2.46 | 0.00 |
| PLPP4 | 5.32 | 0.00 |
| ANOS1 | 3.05 | 0.00 |
| CLSTN2 | 7.66 | 0.00 |
| SOX11 | 3.54 | 0.00 |
| LANCL2 | 2.28 | 0.00 |
| QPCT | 2.90 | 0.00 |
| SLC17A9 | 2.01 | 0.00 |
| VSTM2L | 6.07 | 0.00 |
| USP46 | 2.00 | 0.00 |
| CSMD2 | 2.43 | 0.00 |
| PDCD1LG2 | 2.92 | 0.00 |
| CTHRC1 | 2.15 | 0.00 |
| MYORG | 2.30 | 0.00 |
| HS3ST3A1 | 3.81 | 0.00 |
| DNER | 5.58 | 0.00 |
| PTPRB | 4.14 | 0.00 |
| NRIP3 | 2.48 | 0.00 |
| SLC29A1 | 2.01 | 0.00 |
| PDZD2 | 2.27 | 0.00 |
| ZSWIM4 | 2.26 | 0.00 |
| NPY4R | 3.31 | 0.00 |
| CDH4 | 3.21 | 0.00 |
| MYO7B | 2.52 | 0.00 |
| XRCC4 | 5.65 | 0.00 |
| GRIA3 | 5.05 | 0.00 |
| ST6GAL2 | 24.50 | 0.00 |
| FHOD3 | 2.15 | 0.00 |
| EGR2 | 5.52 | 0.00 |
| CLDN14 | 8.97 | 0.00 |
| PRICKLE1 | 2.12 | 0.00 |
| TIAM1 | 2.90 | 0.00 |
| HBEGF | 3.72 | 0.00 |
| CILP | 32.77 | 0.00 |
| TIMP3 | 3.91 | 0.00 |
| COMP | 22.01 | 0.00 |
| KIRREL3 | 2.26 | 0.00 |
| COL4A4 | 2.72 | 0.00 |
| SCN9A | 4.23 | 0.00 |
| MYOM3 | 6.16 | 0.00 |
| CSRP2 | 2.58 | 0.00 |
| MATN3 | 5.13 | 0.00 |
| DNAJB9 | 2.47 | 0.00 |
| RAB3B | 3.19 | 0.00 |
| MLLT11 | 2.24 | 0.00 |
| CNTN1 | 32.33 | 0.00 |
| LAMC2 | 4.22 | 0.00 |
| EGR1 | 2.16 | 0.00 |
| SYDE2 | 2.08 | 0.00 |
| SLC46A3 | 2.47 | 0.00 |
| RBP1 | 6.11 | 0.00 |
| ULBP2 | 2.22 | 0.00 |
| LIPG | 2.42 | 0.00 |
| ISLR2 | 25.50 | 0.00 |
| EPGN | 8.23 | 0.00 |
| SEMA7A | 4.59 | 0.00 |
| HHAT | 2.85 | 0.00 |
| PPP1R14C | 3.88 | 0.00 |
| DRP2 | 8.22 | 0.00 |
| DSG2 | 2.14 | 0.00 |
| NXPH3 | 2.41 | 0.00 |
| PCDH10 | 3.48 | 0.00 |
| IL11 | 38.88 | 0.00 |
| PTGS2 | 7.64 | 0.00 |
| S1PR5 | 42.63 | 0.00 |
| RBM24 | 4.11 | 0.00 |
| TEK | 4.39 | 0.00 |
| HEYL | 6.41 | 0.00 |
| DYNC1I1 | 5.29 | 0.00 |
| CGB3;CGB1;ENSG00000267335 | 38.22 | 0.00 |
| TNFRSF9 | 2.03 | 0.00 |
| TSPAN13 | 11.70 | 0.00 |
| HIC1 | 2.03 | 0.00 |
| EFHD1 | 2.26 | 0.00 |
| CHST6 | 3.64 | 0.00 |
| TNFSF9 | 2.55 | 0.00 |
| JPH2 | 2.95 | 0.00 |
| NPTX1 | 12.26 | 0.00 |
| UCP2 | 8.05 | 0.00 |
| NFATC2 | 3.74 | 0.00 |
| AFAP1L1 | 2.56 | 0.00 |
| SCD | 2.30 | 0.00 |
| HTR1D | 54.75 | 0.00 |
| KRT81 | 4.92 | 0.00 |
| PKP1 | 3.05 | 0.00 |
| PLEK2 | 5.02 | 0.00 |
| MCAM | 2.53 | 0.00 |
| HYI | 6.07 | 0.00 |
| CD70 | 2.82 | 0.00 |
| HTR2A | 6.27 | 0.00 |
| YRDC | 2.06 | 0.00 |
| GDF10 | 10.85 | 0.00 |
| OSCAR | 2.64 | 0.00 |
| ADAMTS16 | 4.71 | 0.00 |
| ANXA8 | 3.61 | 0.00 |
| MYLK2 | 6.83 | 0.00 |
| ARL4A | 2.18 | 0.00 |
| SHC3 | 2.88 | 0.00 |
| FOSB | 3.47 | 0.00 |
| SLC1A3 | 3.72 | 0.00 |
| TM6SF1 | 3.43 | 0.00 |
| GAS7 | 2.25 | 0.00 |
| SIK1 | 3.31 | 0.00 |
| CSF1R | 2.88 | 0.00 |
| SCGB3A2 | 10.26 | 0.00 |
| LAPTM5 | 2.70 | 0.00 |
| LINGO3 | 4.31 | 0.00 |
| TFPI2 | 2.85 | 0.00 |
| CCNJL | 2.95 | 0.00 |
| CD274 | 2.98 | 0.00 |
| ACTBL2 | 33.79 | 0.00 |
| STRA6 | 3.13 | 0.00 |
| FRMPD3 | 4.14 | 0.00 |
| CILP2 | 3.75 | 0.00 |
| OPCML | 316.18 | 0.00 |
| EMB | 2.75 | 0.00 |
| RASSF7 | 2.05 | 0.00 |
| SCX | 4.09 | 0.00 |
| CGB2 | 6.92 | 0.00 |
| NPY4R2 | 2.53 | 0.00 |
| USP43 | 2.76 | 0.00 |
| AFAP1L2 | 2.61 | 0.00 |
| BCL11A | 4.50 | 0.00 |
| CCDC85A | 5.48 | 0.00 |
| DPT | 2.44 | 0.00 |
| KCNMB1 | 2.66 | 0.00 |
| ZPLD1 | 65.91 | 0.00 |
| TMEM51 | 2.22 | 0.00 |
| DNAJB13 | 4.62 | 0.00 |
| NKAIN4 | 161.63 | 0.00 |
| PLXDC1 | 2.01 | 0.00 |
| BHLHA15 | 2.90 | 0.00 |
| CYS1 | 3.60 | 0.00 |
| POU2F2 | 2.43 | 0.00 |
| PABPC4L | 2.80 | 0.00 |
| CCIN | 2.74 | 0.00 |
| COL20A1 | 104.06 | 0.00 |
| IGF1 | 432.44 | 0.00 |
| FIBCD1 | 6.84 | 0.00 |
| TECTB | 41.99 | 0.00 |
| SERPINA9 | 2.74 | 0.00 |
| PTHLH | 2.63 | 0.00 |
| KCNJ6 | 2.94 | 0.00 |
| CES1 | 3.59 | 0.00 |
| HAS1 | 3.08 | 0.00 |
| ERVMER34-1 | 24.79 | 0.00 |
| KRT79 | 41.13 | 0.00 |
| NIPAL4 | 2.89 | 0.00 |
| P2RX1 | 2.44 | 0.00 |
| ENSG00000285868;FNDC9 | 2.63 | 0.00 |
| PARM1 | 3.38 | 0.00 |
| RNF165 | 5.55 | 0.00 |
| ODAPH | 18.03 | 0.00 |
| KRT4 | 16.82 | 0.00 |
| DOC2B | 6.06 | 0.00 |
| BMP2 | 2.03 | 0.00 |
| PSAPL1 | 2.22 | 0.00 |
| C3orf80 | 3.38 | 0.00 |
| DCLK3 | 15.45 | 0.00 |
| GPRIN2 | 2.50 | 0.00 |
| FOXF2 | 2.37 | 0.00 |
| AGTR1 | 3.25 | 0.00 |
| ALOX5AP | 1152.73 | 0.00 |
| ATP4A | 8.81 | 0.00 |
| TMEM100 | 8.10 | 0.00 |
| BIRC7 | 13.91 | 0.00 |
| SUSD4 | 2.90 | 0.00 |
| SLC22A31 | 4.60 | 0.00 |
| CGB5 | 34.66 | 0.00 |
| DCHS2 | 2.10 | 0.00 |
| KISS1 | 2180.53 | 0.00 |
| LPAR5 | 3.41 | 0.00 |
| IGFBPL1 | 433.27 | 0.00 |
| OLR1 | 504.38 | 0.00 |
| CRTAC1 | 52.06 | 0.00 |
| SLC13A5 | 5.34 | 0.00 |
| ARTN | 2.24 | 0.00 |
| PPP1R14A | 3.34 | 0.00 |
| ZNF474 | 2.88 | 0.00 |
| ACHE | 2.92 | 0.00 |
| PDGFB | 5.50 | 0.00 |
| PADI1 | 2.88 | 0.00 |
| DUSP2 | 2.38 | 0.00 |
| MTUS2 | 2.02 | 0.00 |
| IKZF3 | 122.90 | 0.00 |
| SYT8 | 2.25 | 0.00 |
| TYR | 445.20 | 0.00 |
| KDR | 2.74 | 0.00 |
| MYCL | 3.61 | 0.00 |
| KRT34 | 2.40 | 0.00 |
| NPY1R | 325.39 | 0.00 |
| KANK4 | 117.06 | 0.00 |
| SPINK1 | 726.24 | 0.00 |
| PTPRR | 4.76 | 0.00 |
| CALB2 | 39.31 | 0.00 |
| AMTN | 29.39 | 0.00 |
| MUC7 | 389.37 | 0.00 |
| SPNS3 | 7.47 | 0.00 |
| ENSG00000289697 | 2.82 | 0.00 |
| NKX2-6 | 579.11 | 0.00 |
| FPR1 | 6.05 | 0.00 |
| SLC5A5 | 6.24 | 0.00 |
| PGA4;PGA5 | 33.26 | 0.00 |
| EGF | 2.04 | 0.00 |
| PDE4C | 10.80 | 0.00 |
| PKP2 | 2.20 | 0.00 |
| KRT17 | 5.83 | 0.00 |
| ATP2B3 | 3.03 | 0.00 |
| GBX2 | 5.51 | 0.00 |
| NECTIN4 | 5.19 | 0.00 |
| PGA3 | 64.86 | 0.00 |
| TMPRSS3 | 150.13 | 0.00 |
| KCNMB4 | 7.32 | 0.00 |
| CAPNS2 | 4.13 | 0.00 |
| CCNA1 | 8.61 | 0.00 |
| CTAGE1 | 3.09 | 0.01 |
| ADARB2 | 2.89 | 0.01 |
| FPR3 | 249.07 | 0.01 |
| SULT1B1 | 4.29 | 0.01 |
| OR2F1 | 142.82 | 0.01 |
| ASB18 | 4.76 | 0.01 |
| PRDM1 | 11.56 | 0.01 |
| TPSG1 | 496.64 | 0.01 |
| SLC30A2 | 4.83 | 0.01 |
| CAPN13 | 119.06 | 0.01 |
| P2RY14 | 2.12 | 0.02 |
| PLD5 | 62.48 | 0.02 |
| GJA5 | 7.28 | 0.02 |
| TYRP1 | 25.57 | 0.02 |
| KMO | 4.63 | 0.02 |
| WNT7B | 3.07 | 0.02 |
| ELF3 | 2.05 | 0.02 |
| DCSTAMP | 4.88 | 0.02 |
| RASL12 | 6.11 | 0.02 |
| OR5P2 | 2.30 | 0.03 |
| DUSP26 | 357.20 | 0.03 |
| TNFRSF11A | 2.24 | 0.03 |
| NPPB | 19.53 | 0.03 |
| LGALS9C | 3.46 | 0.04 |
| KEL | 4.60 | 0.04 |
| SAA2 | 2.15 | 0.04 |
| FGD2 | 43.53 | 0.05 |

LF, ligamentum flavum.
